# Intersecting Inequities in Ischemic Heart Disease among U.S. Older Adults: Trends by Nativity, Sex & Race, 2005–19

**DOI:** 10.1093/geroni/igaf122.4147

**Published:** 2025-12-31

**Authors:** Stuti Das

**Affiliations:** Boston University, Boston, Massachusetts, United States

## Abstract

Ischemic heart disease (IHD) remains a leading cause of morbidity and mortality in later life, with prevalence rising steeply after midlife. While disparities by sex and race/ethnicity are well documented, less is known about how these inequities evolve at the intersection of nativity, sex, and race/ethnicity among U.S. adults aged 45 and older. Using harmonized data from the Integrated Public Use Microdata Series of the National Health Interview Survey (IPUMS NHIS, 2005–2019), I examine trends in self-reported IHD diagnoses. Joinpoint regression identifies inflection points in age-standardized prevalence across subgroups defined by nativity, sex, and race/ethnicity, followed by moderation analyses to assess how these identities jointly shape disease risk. Findings reveal that foreign-born adults consistently report lower IHD prevalence than their U.S.-born peers across sex and racial/ethnic groups. However, this immigrant health advantage is not uniform. Native-born White women are the only group to experience a sustained decline in IHD over time, while the nativity gap is widest among non-Hispanic Black and Asian men. Some immigrant subgroups, such as Asian immigrant women, show slight increases, though still below native-born levels. These findings underscore the importance of an intersectional, equity-oriented lens for understanding disparities in cardiovascular aging and for informing more inclusive public health strategies. As the U.S. population grows older and more diverse, it is critical to examine how migration, racialization, gender, and aging interact to shape chronic disease risk and resilience.

